# Pseudo-apical hypertrophic cardiomyopathy phenotype in Takotsubo cardiomyopathy

**DOI:** 10.1007/s10554-025-03577-6

**Published:** 2025-12-06

**Authors:** Mohamed Elzoghby, Anna B. Reid, Gaetano Nucifora

**Affiliations:** 1 Northwest Heart Centre, Cardiac Imaging Unit, Wythenshawe Hospital, Manchester University NHS Foundation Trust, Manchester, UK; 2 Division of Cardiovascular Sciences, School of Medical Sciences, Faculty of Biology, Medicine and Health, Manchester Academic Health Science Centre, University of Manchester, Manchester, UK

**Keywords:** Apical hypertrophic cardiomyopathy, Cardiac magnetic resonance, Differential diagnosis, Myocardial oedema, Takotsubo cardiomyopathy, T2 mapping

## Abstract

Takotsubo cardiomyopathy can exhibit transient apical wall thickening during recovery, leading to a phenotypic overlap with apical hypertrophic cardiomyopathy. This pseudo-hypertrophic appearance is due to reversible myocardial oedema rather than genuine sarcomeric hypertrophy. We present a case demonstrating this diagnostic challenge and emphasising the importance of serial cardiac magnetic resonance imaging with tissue characterisation.

## Case information

**Fig. 1:**
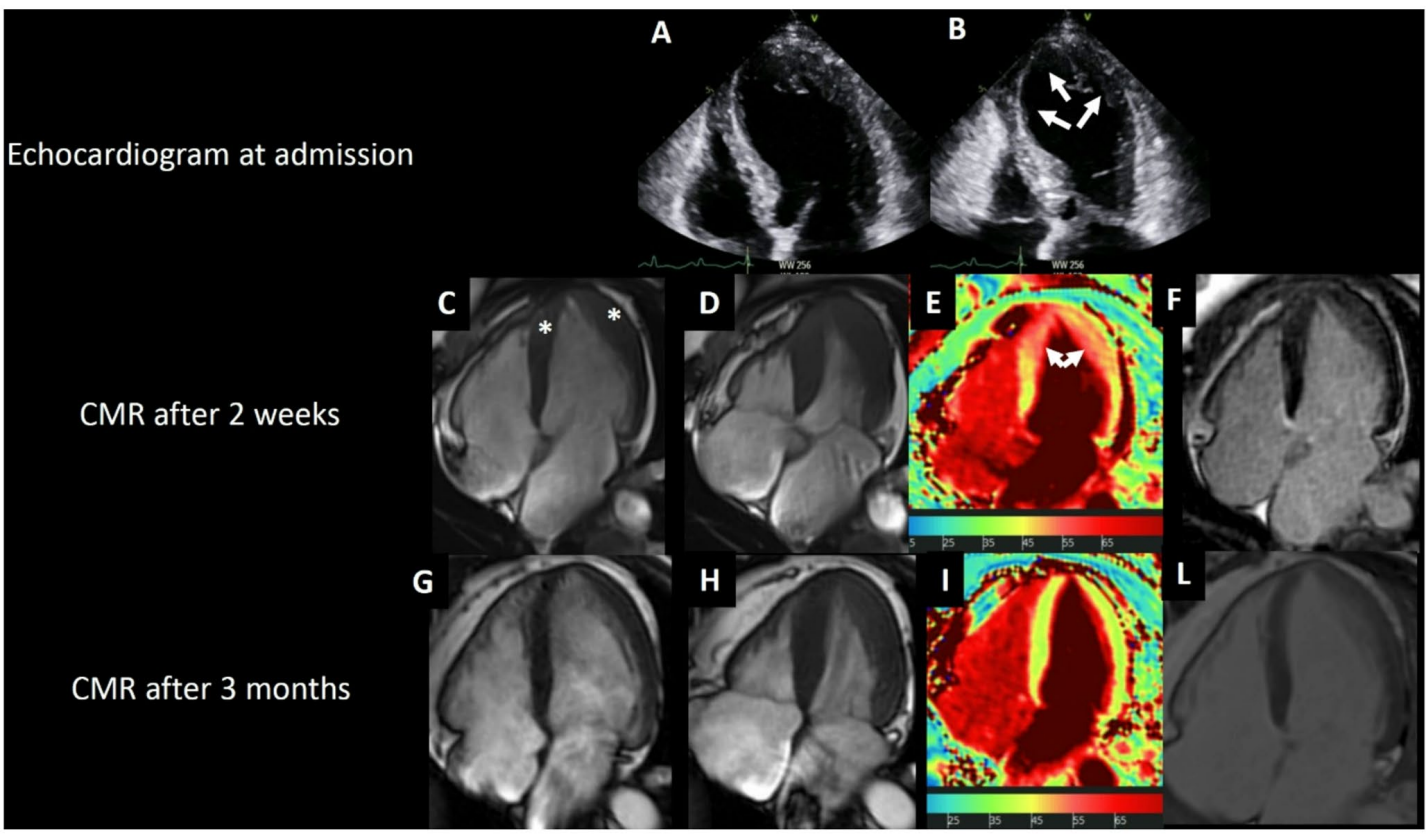
Temporal evolution of pseudo-apical hypertrophic cardiomyopathy phenotype in Takotsubo cardiomyopathy. Panels A-B: Echocardiogram performed at admission. Four-chamber end-diastolic (**A**) and end-systolic (**B**) frames showing mid-apical left ventricular akinesia with apical ballooning. Panels C-F: Cardiac magnetic resonance imaging performed two weeks after presentation. Four-chamber cine CMR images in end-diastole (**C**) and end-systole (**D**) show normalised ejection fraction with increased apical wall thickness (*; 13 mm) creating a pseudo-apical hypertrophic cardiomyopathy appearance. T2 mapping (**E**) demonstrates elevated T2 values (arrows; up to 60 ms) in the apical segments, indicating myocardial oedema and inflammation. Four-chamber late gadolinium enhancement imaging (**F**) shows no myocardial scar or replacement fibrosis. Panels G-L: Follow-up cardiac magnetic resonance imaging at 3 months. Four-chamber cine CMR images in end-diastole (**G**) and end-systole (**H**) show complete normalisation of myocardial thickness with preserved normal ejection fraction. T2 mapping (**I**) indicates normalised T2 values, signifying resolution of myocardial oedema. Late gadolinium enhancement imaging (**L**) continues to reveal absence of myocardial scar

A 55-year-old woman presented with chest pain and anterolateral ST-elevation. Coronary angiography showed normal arteries. Echocardiography revealed moderate to severe left ventricular dysfunction with apical ballooning (Panels A-B), consistent with Takotsubo cardiomyopathy (Fig. 1).

CMR at two weeks showed a normalised left ventricular ejection fraction but increased apical thickness (Panels C-D) with elevated T2 values (Panel E), indicating myocardial oedema [1]. No late gadolinium enhancement (LGE) was observed (Panel F). Follow-up CMR at three months documented complete normalisation of thickness and T2 values (Panels G-I), confirming reversible changes.

The pseudo-apical HCM phenotype results from catecholamine-mediated injury, leading to reversible interstitial oedema without genuine sarcomeric hypertrophy [2]. The presence of elevated T2 values indicating inflammation, along with the absence of LGE, distinguishes this from true HCM.

Recognition of pseudo-apical HCM phenotype in Takotsubo prevents misdiagnosis. Apparent apical thickening may mimic HCM during subacute recovery. Serial CMR with tissue characterisation distinguishes reversible oedema from true hypertrophic cardiomyopathy, avoiding inappropriate long-term management [3].

## Data Availability

No datasets were generated or analysed during the current study.
